# Physical activity, sedentary behavior and total wellness changes among sedentary adults: a 4-week randomized controlled trial

**DOI:** 10.1186/1477-7525-11-183

**Published:** 2013-10-29

**Authors:** Faisal A Barwais, Thomas F Cuddihy, L Michaud Tomson

**Affiliations:** 1 School of Exercise and Nutrition Sciences, Institute of Health and Biomedical Innovation, Queensland University of Technology, Brisbane, Australia; 2 Department of Physical Education and Sports, Umm Al-Qura University, Makkah, Saudi Arabia; 3 School of Education and Professional Studies, Griffith University, Brisbane, Australia

**Keywords:** Sedentary behavior, Wellness evaluation of lifestyle (WEL), IPAQ, 7-day SLIPA Log

## Abstract

**Background:**

The construct of total wellness includes a holistic approach to the body, mind and spirit components of life. While the health benefits of reducing sedentary behavior and increasing physical activity are well documented, little is known about the influence on total wellness of an internet-based physical activity monitor designed to help people to achieve higher physical activity levels.

**Purpose:**

The purpose of this four-week, personal activity monitor-based intervention program was to reduce sedentary behavior and increase physical activity levels in daily living for sedentary adults and to determine if these changes would also be associated with improvement in total wellness.

**Methods:**

Twenty-two men and 11 women (27 years ± 4.0) were randomly assigned to either an intervention (n = 18) or control group (n = 15). The intervention group interacted with an online personal activity monitor (Gruve Solution™) designed to reduce sedentary time and increase physical activity during activities of daily living. The control group did not interact with the monitor, as they were asked to follow their normal daily physical activities and sedentary behavior routines. The Wellness Evaluation of Lifestyle (WEL) inventory was used to assess total wellness. Sedentary time, light, walking, moderate and vigorous intensity physical activities were assessed for both intervention and control groups at baseline and at week-4 by the 7-day Sedentary and Light Intensity Physical Activity Log (7-day SLIPA Log) and the International Physical Activity Questionnaire (IPAQ).

**Results:**

Significant increases in pre-post total wellness scores (from 64% ± 5.7 to 75% ± 8.5) (t _(17)_ = -6.5, *p* < 0.001) were observed in the intervention group by the end of week four. Intervention participants decreased their sedentary time (21%, 2.3 hours/day) and increased their light (36.7%, 2.5 hours/day), walking (65%, 1057 MET-min/week), moderate (67%, 455 MET-min/week) and vigorous intensity (60%, 442 MET-min/week) physical activity (all *p* < 0.001). No significant differences for total wellness were observed between the groups at baseline and no pre-post significant differences were observed for any outcome variable in the control group.

**Conclusion:**

Total wellness is improved when sedentary, but sufficiently physically active adults, reduce sedentary time and increase physical activity levels (i.e. light, waking, moderate and vigorous).

## Introduction

In many parts of the world, lifestyles have become increasingly sedentary in the home, at work and during leisure time, particularly given the increasing popularity of computer usage, video game playing and television viewing [1]. In general, sedentary behavior and light-intensity physical activity behaviors have become increasingly common in adults. There is substantial evidence showing that adults spend most of their waking hours either in sedentary or in light-intensity physical activities [2].

An increasing body of evidence suggests that sedentary behaviors are associated with poor health outcomes. Sedentary time, independent of the time spent in moderate to vigorous intensity physical activity, is associated with health risks including type 2 diabetes [3], cardiovascular disease [3,4], metabolic syndrome [5], weight gain [6,7] and obesity [8,9]. Consequently, increased attention is being paid to the development of intervention methods that focus on reducing sedentary time and increasing physical activity levels for the purpose of improving overall health [10,11]. Little is currently known about the effect of such interventions on total wellness, a concept which encompasses much more than physical health. Wellness has been described as, “. . . a way of life oriented toward optimal health and wellbeing, in which body, mind and spirit are integrated by the individual to live life more fully within the human and natural community. Ideally, it is the optimum state of health and well-being that each individual is capable of achieving.” (p. 252) [12]. In other words, wellness is a term that encompasses an individual’s outlook on life, including their perceptions of personal physical activity, happiness, learning, society, work and spirituality [13]. Wellness involves interaction among six or more dimensions (e.g., physical, occupational, social, spiritual, intellectual and emotional) that are enmeshed, related and, when balanced properly, provide the individual with optimal health or “high-level wellness” [14,15]. Because all wellness dimensions are interrelated, a change in one area causes or contributes to changes in other areas, which subsequently influence total wellness scores [16]. To some researchers in public health, the physical dimension represents a person’s ability to function effectively in meeting the demands of the day's work and to use free time effectively; this includes participation in regular appropriate physical activity [17] and possession of useful motor skills [13]. Given that significant numbers of adults continue to be sedentary, it may prove beneficial to determine if an intervention designed to reduce sedentary behavior and increase physical activity can influence total wellness scores. This may enable public health advocates to formulate broader messages about the benefits of reductions in sedentary behaviors and increases in light and/or moderate to vigorous physical activity. The purpose of the current study, therefore, was to evaluate participation in a four-week, online, personal activity monitor-based intervention for sedentary but sufficiently active adults. The intervention was intended to reduce sedentary behavior and increase physical activity in daily living activities, and this study sought to determine whether such changes were associated with improvement in total wellness.

## Methods

### Design overview

This study used a pre–post randomized control trial (RCT). At baseline, a computer random number generator was employed to allocate participants into either an intervention (n = 18) or control (n = 15) group. The intervention group engaged with the personal activity monitor during the four-week program. The control group did not interact with the monitor and were asked to follow their normal, daily lifestyle patterns. Written informed consent was obtained from all participants and the study was approved by the Human Research Ethics Committee of Queensland University of Technology. Participation was completely voluntary and participants were informed that they could withdraw at any time during the intervention.

### Participants

Thirty-three adults (22 men, 11 women; mean age 27 yrs. ± 4 yrs.) were recruited to participate in the study through advertisements in local newsletters, flyers and emails at a metropolitan university in Brisbane, Australia, during October/November 2012. Pre-screening using a self-report questionnaire was used to determine eligibility in relation to sedentary behavior. Only those who reported a high total sitting time, defined as spending > 7 hours per day, were invited to participate in the study [18,19]. The sample size for this study was set at a minimum of 32 by using G*Power V.1.1.3 software set for F-test analysis of variance (ANOVA). Power was calculated at 0.8, Alpha level was set at *p* < .05 and an effect size was set at 0.5 for the two groups (intervention and control).

### Intervention (online personal activity monitor) group

Participants in the intervention group interacted with an online personal activity monitor (Gruve Solution™ MUVE, Inc., USA). The device was designed to motivate a reduction in sedentary behavior and increase physical activity in the activities of daily living. The Gruve Solution is an activity-based wellness approach built around the concept of non-exercise activity thermogenesis (NEAT) [20]. NEAT is comprised of low energy expenditure during daily activities such as standing, walking, sitting and fidgeting, all of which are activities that are not considered planned physical activity in a person’s daily life [21]. Changing one’s postural position from seated to standing or engaging in light ambulation has been shown to significantly increase energy expenditure [21]. The monitor is a tri-axial accelerometer system that tracks time spent on daily sedentary, light-, moderate-, and vigorous-intensity physical activity via a wearable device and an accompanying online service. It monitors a participant’s daily physical activity at 20 Hz and stores the minute data on the device for later uploading to the interactive online software through a Universal Serial Bus (USB) port. These data subsequently provide the user with an easy-to-understand visualization of daily activity patterns. Goal-setting features are activated alongside simple graphs and charts to enhance the self-monitoring of energy expenditure. An indicator (a halo bar) on top of the device also highlights the user’s progress towards their daily goal. When palpated throughout the day, the indicator bar provides a Light-Emitting Diode (LED) color corresponding to the user’s progress towards their daily activity goal. For example, at the beginning of the day the light bar is red but, as the day progresses, if the user has been sufficiently active, then the color progresses to yellow to orange to blue and, finally, to green. The green light indicates that the daily activity goal has been achieved. Research has shown that the Gruve monitor is accurate both when measuring energy expenditure at seven velocities in the laboratory and during activities of daily living [22,23]. In one recent study, the Gruve Solution™ was one of three devices used by the STAND project (Sedentary Time and Diabetes, 2011) to reduce sedentary behavior in younger adults [24]. The research team involved in the study suggested that the Gruve was the most appropriate self-monitoring device for reducing sedentary time.

During this study’s four-week program, participants in the intervention group wore the monitor on a daily basis, both on weekdays and weekends during activities daily of living (except when sleeping, bathing or swimming). To increase their motivation, participants were encouraged to achieve their daily monitor goals as recommended by Gruve Solution™ guidelines [20] and view their daily online homepages. Weekly motivational emails from the online system were sent to participants when they achieved their goals. The emails were designed to encourage the participants to continue to be more active than their habitual physical activity level as determined during the baseline week.

### Outcome measures

#### Sedentary and light intensity physical activity

Participants were assessed at baseline and at the end of the 4-week involvement. Daily sedentary and light intensity physical activities for both intervention and control groups were measured using the 7-day Sedentary and Light Intensity Physical Activity Log (7-day SLIPA Log) [25]. The 7-day SLIPA Log is a 23-item instrument that collects information about sedentary behavior and light intensity physical activity across typical daily life situations. The validity of the 7-day SLIPA Log was validated against an ActiGraph GT3X accelerometer for seven consecutive days with a cut-point for sedentary time defined as <100 counts per minute (cpm) and light-intensity physical activities as 100–1951 cpm. The correlation between the 7-day SLIPA Log and the GT3X was *r* =0.86, *p* < 0.001 for sedentary time and *r* =0.80, *p* < 0.001 for light intensity physical activity which was found to be acceptable [25].

Participants in both the intervention and control groups of the instant study were asked to complete the 7-day SLIPA Log on a daily basis by indicating how many hours and minutes they spent in sedentary behavior and light-intensity physical activity on each of four, 'daily life’ behavioral domains during the previous day (12:00 a.m. to 11:59 p.m.).

#### Walking, moderate and vigorous intensity physical activity

In order to assess the participants’ physical activity levels at baseline and post-intervention (end of the 4th week), the short version of the International Physical Activity Questionnaire (IPAQ) was used. The IPAQ has been shown to be reliable and valid in a study involving 12 countries [26]. It assesses physical activity levels by asking participants to answer questions regarding the frequency (days per week), duration (in hours and minutes) and level of intensity (walking, moderate and vigorous) of physical activity during the previous seven days. The IPAQ is scored by using the Metabolic Equivalent of Task (MET) method, in which different activities and levels of intensity are assigned different MET estimates. In this study, total MET-minutes per week were calculated separately for walking, moderate and vigorous intensity activities [17].

#### Wellness evaluation of lifestyle (WEL) inventory

Wellness was measured using the online version of the WEL inventory, which was developed for institutions to simplify the collection and evaluation of data [27]. Derived from the Wheel of Wellness theoretical model, the WEL was developed as a method for describing wellness behaviors that encompass factors related to the participants’ body, mind, and spirit [12]. The Wheel model includes five major life tasks, which are supported by empirical data and posit important characteristics of that are considered to be central to an individual’s healthy functioning [27]. These life tasks include spirituality, work and leisure, friendship, love, and self-regulation. The life task of self-regulation (viewed as functioning much like the spokes in a wheel to give strength to the wheel as a whole) includes twelve additional components: (1) sense of worth, (2) sense of control, (3) realistic beliefs, (4) emotional awareness and coping, (5) intellectual stimulation, problem solving and creativity, (6) sense of humor, (7) exercise/physical activity, (8) nutrition, (9) self-care, (10) stress management, (11) gender identity, and (12) cultural identity [27]. The WEL consists of 103 items represented as self-statements to which respondents reply using a five-point Likert- scale with the following options: (a) strongly agree, (b) agree, (c) undecided or neutral, (d) disagree and (e) strongly disagree. A score is computed by summing the 103 items and producing a total score (range = 103 to 515). For ease of interpretation, the total score is divided by the total points possible (515) to yield a percentage value. The WEL has been shown to demonstrate construct validity and reliability in previous research [12,28] and has been used to assess wellness among adults [29].

Participants in both intervention and control groups of this study were asked to complete the WEL at baseline and the end of the 4-weeks. Total wellness was determined by calculating a total percentage of the sum of the five life tasks of spirituality, work and leisure, friendship, love, and self-regulation.

### Statistical analyses

Data were analyzed in 2013 using SPSS statistical software version 21.0 for Windows (SPSS Inc., Chicago, IL). Descriptive data were expressed as means and SDs (95% confidence intervals). Paired sample t-tests were conducted to compare intervention and control groups at baseline and at the end of the 4-week involvement on outcome measures of the 7-day SLIPA Log and IPAQ scores.

To determine whether changes in total wellness differed for the intervention group and control group, MANOVA was used to analyze the effect of Treatment (intervention vs. control group), Time (baseline vs. the end of the 4-week intervention) and Group by Time (interaction). A follow-up univariate analysis was conducted to identify group differences. Paired sample t-tests were calculated to evaluate within-group changes. Effect sizes for mean differences were expressed as Cohen’s *d* (difference in means divided by the standard deviation of the difference) and interpreted as small, moderate, or large based on values of 0.2, 0.5, and 0.8, respectively [30].

## Results

The study sample (N = 33) averaged 27 years of age, while 63% were office workers and 37% were full-time students. Participants had both high total sitting time (9.0 ± .9 hours/day sitting) and sufficient physical activity (≥ 600 MET-min/week) as determined by a self-report questionnaire (IPAQ). At baseline, there were no significant differences found either within or between the intervention and control group on any of the demographics or variables of interest. (See Table 1).

**Table 1:** Demographics of the study population

	**Intervention group**			**Control group**		
	**Total (n = 18)**	**Male (n = 12)**	**Female (n = 6)**	**Total (n = 15)**	**Male (n = 10)**	**Female (n = 5)**
Age (years)	29.0 ± 4.4	28.7 ± 4.9	29.5 ± 3.5	26.4 ± 3.0	26.1 ± 2.7	27.2 ± 3.8
Height (cm)	171.9 ± 9.8	174.1 ± 10.8	167.6 ± 6.1	170.4 ± 8.0	173.8 ± 6.7	163.8 ± 6.1
Weight (Kg)	78.3 ± 20.6	84.6 ± 20.7	65.6 ± 14.5	77.7 ± 24.4	83.2 ± 28.5	66.8 ± 6.3

Differences between the intervention and control group at baseline and at the end of the 4-week intervention in time spent on sedentary activity and light intensity physical activity, walking, moderate and vigorous intensity physical activity are illustrated in Table 2. Paired-sample t-tests indicated a significant decrease in sedentary time from baseline to the end of the 4-weeks for the intervention group. Initially, sedentary time was 10.9 ± 1.9 hours/day; after the intervention, it dropped to 8.6 ± 1.7 hours/day [*t* (17) = 7.7, *p* < 0.001]. In addition, the intervention group had a significant increase in time spent on light-intensity physical activity from baseline to the end of the 4-weeks. The change was from 4.3 ± 2.0 hours/day to 6.8 ± 1.7 hours/day [*t* (17) = -7.0, *p* < 0.001]. For the control group, no significant differences were observed between pre (10.7 ± 2.5 hours/day) and post (11.2 ± 1.5 hours/day) sedentary time [*t* (14) = -.60, *p* = 0.55)] or between pre (3.6 ± 1.9 hours/day) and post (3.2 ± 1.7 hours/day) light intensity physical activity [*t* (14) = 1.2, *p* = 0.24].

**Table 2:** Pre-post differences between intervention and control groups on the 7-day SLIPA Log and IPAQ scores

	**Intervention group**					**Control group**			
	**Baseline**	**Week 4**	***t***	***p*-value**	**Cohen**	**Baseline**	**Week 4**	***t***	***p*-value**
**Sedentary**‡	10.9 ±1.9	8.6 ± 1.7	7.7	*p* < .01	***d *** **=1.30**	10.7 ± 2.5	11.2 ±1.5	-.60	*p* =0.55
**Light** ‡	4.3 ± 2.0	6.8 ± 1.7	-7.0	*p* < .01	***d *** **=2.06**	3.6 ± 1.9	3.2 ± 1.7	1.2	*p* =0.24
**Walking †**	568 ± 531.5	1625 ± 553.8	-7.2	*p* < .01	***d *** **=2.0**	537 ± 299.0	483 ± 175.9	-.71	*p* =0.49
**Moderate**	194 ± 225.3	649 ± 494.9	-4.3	*p* < .01	***d *** **=1.22**	180 ± 207.5	192 ± 221.3	-.13	*p* =0.89
**Vigorous†**	291 ± 495.8	733 ± 829.3	-3.3	*p* < .01	***d *** **=0.67**	173 ± 298.8	193 ± 291.2	-.32	*p* =0.74

‡ 7-day SLIPA Log variable **(**hours/day) † IPAQ variable (MET-minutes/week).

A Cohen *d* value of **0.8** or greater indicates a large effect size.

Paired-sample t-tests indicated a significant increase in IPAQ scores for the intervention group between baseline (568 ± 531.5 MET-min/week) and at the end (1625 ± 553.8 MET-min/week) of the 4-week intervention for walking [*t* (17) = -7.1, *p* < 0.001]. IPAQ scores for moderate intensity physical activity also increased from pre (194 ± 225.3 MET-min/week) to post (649 ± 494.9 MET-min/week) measurement [*t* (17) = -4.3, *p* < 0.001]. This also occurred for vigorous intensity physical activity (pre = 291 ± 495.8 MET-min/week; post = 733 ± 829.3 MET-min/week) [*t* (17) = -3.3, *p* < 0.001]. (See Table 2). No significant differences were observed for the control group between baseline and at the end of the 4-week intervention for walking (pre = 537 ± 299.0 MET-min/week; post = 483 ± 175.9 MET-min/week) [*t* (14) = .71, *p* = 0.490], moderate intensity physical activity (pre = 180 ± 207.5 MET-min/week; post = 192 ± 221.3 MET-min/week) [*t* (14) = -.13, *p* =0.892] and vigorous intensity physical activity (pre = 173 ± 298.8 MET-min/week; post = 193 ± 291.2 MET-min/week) [*t* (14) = -.32, *p* =0.749] (see Table 2).

**Figure 1:**
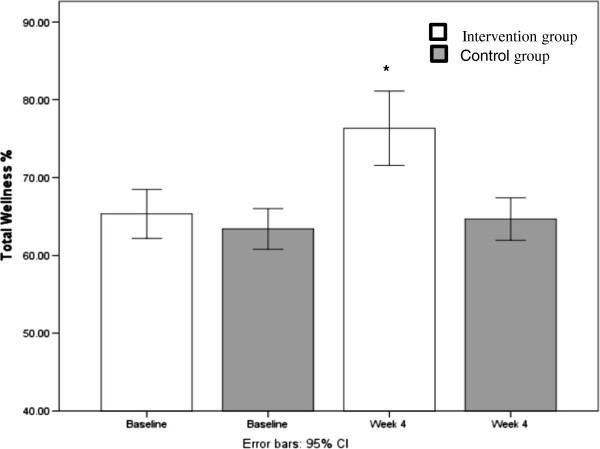
**Total wellness percentage score increase from baseline to week 4.** *(*p* < 0.001).

Comparison of total wellness percentage scores between the intervention and control group are shown in Figure 1. The 'Time’ variable represents pre-post conditions (baseline vs. the end of the 4-week intervention) and the 'Treatment’ variable represents either intervention or control group. A repeated-measures MANOVA indicated a significant main effect for Time (Wilks' Lambda = .568), [*F*(1,31) = 23.5, *p <* .001, *partial η*^2^ = .432]; however, this main effect was qualified by a significant Time x Treatment interaction (Wilks' Lambda = .680), [*F*(1,31) = 14.6*, p <* .001, *partial η*^2^ = .320]. Univariate tests were conducted to examine this interaction in more detail and revealed a significant Time × Treatment effect on total wellness [*F*(1, 31) = 9.5, *p* < .001, *partial* η^2^ = .235]. Paired-sample t-tests were employed to further investigate the significant interaction between intervention and control groups at baseline and the end of the 4-week intervention (See Table 3). Large effect sizes may be observed in most of the life tasks and in many of the discrete scales. The exercise/physical activity scale displayed the largest effect size (*d* = 3.08) and was the major contributor to the life task of 'self-regulation’. Results indicated a significant increase in pre to post total wellness scores for the intervention group (pre = 64% ± 5.7; post = 75% ± 8.5), [*t* (17) = -6.5, *p* < 0.001]. No significant difference between pre-post total wellness scores were observed for the control group (pre = 63% ± 4.7; post = 64% ± 4.9), [*t* (17) = -.68, *p* = .50].

**Table 3:** Pre-post differences between intervention and control groups on the Wellness Evaluation of Lifestyle (WEL)

	**Intervention group**					**Control group**			
**Variable**	**Baseline**	**Week-4**				**Baseline**	**Week-4**		
**WEL Scale**	Mean%	Mean%	***t***	***p***	Cohen	Mean%	Mean%	***t***	***p***
*Sense of worth*	64	79	*-3.9*	*p* < .001	*d* =1.20	66	66	-.06	*p* =0.95
*Sense control*	73	84	*-3.1*	*p* < .001	*d* =0.62	66	70	-1.0	*p* =0.33
*Realistic beliefs*	61	65	*-1.0*	*p* = 0.32	*d* =0.34	51	55	-.99	*p* =0.33
*Emotional responsiveness*	65	86	*-4.0*	*p* < .01	*d* =1.66	64	68	-.75	*p* =0.46
*Intellectual stimulation*	71	78	-2.7	*p* < .05	*d* =0.81	68	72	-1.3	*p* =0.18
*Sense humor*	64	78	-2.4	*p* < .05	*d* =0.90	65	69	-.78	*p* =0.44
*Nutrition*	66	85	-4.3	*p* < .001	*d* =1.25	60	56	.52	*p* =0.61
*Exercise/physical activity*	58	97	-8.9	*p* < .001	*d* =3.08	54	53	.20	*p* =0.84
*Self care*	83	81	.55	*p* = 0.77	*d* =0.19	77	76	.47	*p* =0.64
*Stress management*	54	64	-2.1	*p* < .05	*d* =0.72	54	61	-1.2	*p* =0.23
*Gender identity*	70	73	-.79	*p* = 0.43	*d* =0.27	71	67	.79	*p* =0.44
*Cultural identity*	73	71	.44	*p* = 0.66	*d* =0.12	65	71	-1.3	*p* =0.19
**LIFE TASKS**									
Total self-regulation†**‡**	66	78	-6.0	*p* < .001	*d* =1.58	63	65	-.88	*p* =0.39
Spirituality†	50	56	*-2.9*	*p* < .001	*d* =0.62	56	59	*-.77*	*p* =0.44
Work†	63	71	-2.9	*p* < .001	*d* =0.80	65	61	1.0	*p* =0.30
Leisure†	53	71	-4.3	*p* < .001	*d* =1.22	57	61	-.90	*p* =0.38
Friendship†	72	80	-2.4	*p* < .05	*d* =0.82	71	70	.19	*p* =0.85
Love†	65	72	-2.4	*p* < .05	*d* =0.37	67	66	.23	*p* =0.81
**Total wellness**	64	75	-6.5	*p* < .001	*d* =1.25	63	64	-.68	*p* =0.50

†Total wellness was determined by calculating the percentage scores from the life tasks, including spirituality, self-regulation, work and leisure, friendship, and love. ‡ Total Self-Regulation was determined by calculating the percentage scores from (*Sense of Worth* through *Cultural Identity* scales*).* A Cohen *d* value of **0.8** or greater indicates a large effect size.

## Discussion

The main findings of this study indicate that the intervention group (who used the online personal activity monitor) decreased sedentary time by 21.8% and increased walking, light-, moderate- and vigorous-intensity physical activity. The effect size (*d =* 1.30) for this pre-post reduction of 2.4 hours in daily sedentary time may be considered large. Intervention group participants significantly increased light intensity activity by 36.7% or 2.5 hours/day); walking by 65%, or 1057 MET-min/week) moderate intensity activity by 67%, or 455 MET-min/week; and vigorous intensity activity by 60%, or 442 MET-min/week. Effect sizes (see Table 2) for these changes ranged from large to medium (*d =* 2.1, *d =* 2.0, *d =* 1.22 and *d =* 0.67, respectively). Several studies have shown that replacing sedentary time with equal amounts of light-, moderate- and vigorous-intensity physical activity is associated with better physical health and improved overall health benefits such as reducing risk of type 2 diabetes [31], cardiovascular disease and premature mortality [32,33]. Moreover, participants from healthy populations who engaged in higher physical activity levels generally report a better of quality of life [34]. Our investigation also found a significant pre-post difference for the intervention group in time spent on light-intensity physical activity. The large effect size (2.06) for this pre-post difference in the intervention group is not surprising given that the Gruve monitor was developed to promote daily NEAT activities, which are composed mainly of energy expenditures related to daily physical activity of light-to-moderate intensity [35].

A unique finding of this study is that there was a significant increase in total wellness scores for the intervention group but not for the control group. Myers and Sweeney [16] propose that all wellness dimensions are interrelated and a change in one area causes or contributes to changes to other areas, which influences total wellness scores. The results of this research provide further support for the notion that wellness has several dimensions, including the physical dimension. A novel aspect of this intervention was that it provided both instant and online feedback to the participants, in the physical dimension. This feedback was visually informative (i.e. it provided a figure which summarized the previous 24 hours of activity as light, moderate and vigorous physical activity as well as sedentary time. Each of these components was displayed as a different color. Consequently, the overall result in the physical experience appears to be a beneficial one. The personalized website may have been an important component also in the significant difference observed (and large effect size) on the physical responsiveness scale. Meaningful changes pre-post scores, on many scales and three of five life tasks (see Table 3) were noted for the intervention group but not for the controls. This difference may be especially observed in the Self-Regulation life task (1.58). Myers, Witmer and Sweeney [27] noted that this life task is critical to the dynamic interaction between an individual’s various life contexts. The pre-post improvement in total wellness scores for the intervention group are in line with previous research that found higher volumes of physical activity and leisure time activity were associated with higher perceived physical and psychological wellness scores and participants with higher volumes of physical activity had greater overall perceived wellness scores [36].

The present study adds to the knowledge base in this area of research by showing that an intervention program aimed at decreasing sedentary behaviors and increasing physical activity levels for sedentary but sufficiently physically active adults may be not only associated with beneficial health outcomes [33], but also an increase in total wellness scores. In this sense, reducing sedentary behaviors and/or engaging in light-, moderate- or vigorous-intensity physical activity may be a valuable approach to improving total wellness.

The present study was one of the first trials to assess the efficacy of using an online personal activity monitor to reduce sedentary behavior, increase physical activity levels and improve total wellness. As a part of the inclusion criteria for this study, behavior status for all participants was determined as sedentary via the IPAQ. One limitation of this study is the relatively small sample size, which consisted of office workers or university students. In addition, the data on sedentary behavior and physical activity were collected using a self-report, thus recall bias may exist. What is currently unknown, however, is the effect of the feedback from the online physical activity monitor on total wellness scores. Does it have a direct positive effect on total wellness or is it a psychological facilitator of enhanced physical activity and reduced sedentariness, which then leads to an increase in total wellness scores? Additional research is needed to explore potential wellness benefits of longer-term reductions (e.g. several months) in measured sedentary time. Further studies should include larger sample sizes with more representative age ranges.

Informed strategies for public health and wellness specialists may also be identified when more in-depth analyses of the information in the WEL data are attempted. A more complete picture of both individual strengths and opportunities may become evident when examined alongside a measure of the five “life tasks” and “subtasks”. These data, potentially available within the Wheel of Wellness output and the subdivision of sedentary time across each of the four daily life domains (work, transportation, home and leisure time), need to be examined. It may be that, by using objective and subjective measurement tools, future studies will attempt to determine where reductions in sedentary time across the four daily life domains are both achievable and desirable for wellness “life tasks and subtasks”. Future research would be enhanced by more process evaluation, such as qualitative interviews with the participants both during and after the intervention. These should be designed to elucidate the nature of the participants’ experience relative to the functioning of the Gruve package.

## Conclusion

This study provides important information for enhancing understanding of the associations between decreased sedentary behavior, increased physical activity levels and increases in total wellness scores among sedentary adults. Total wellness and many “life tasks and subtasks” appear to be capable of being significantly enhanced in the relatively short period of 4 weeks. Such knowledge is essential in the development of public health initiatives that aim to increase the wellness of the population via enhanced physical activity and reduced sedentary behaviors. The online monitor method has demonstrated potential for influencing sedentary adults to adopt healthful lifestyle changes.

## Competing interests

The author declares that they have no competing interests.

## Authors’ contributions

FAB performed all work pertaining to this manuscript. TFC supervised the research, helped analyze the data and revised the manuscript. LMT participated in literature review, and revised the manuscript. All authors revised the text critically for important intellectual content and read and approved the final manuscript.
